# Cardiorespiratory performance and locomotor function of patients with anorectal malformations

**DOI:** 10.1038/s41598-021-98368-z

**Published:** 2021-09-23

**Authors:** Christoph Arneitz, Jana Windhaber, Christina Flucher, Paolo Gasparella, Eva Amerstorfer, Andrea Huber-Zeyringer, Christoph Castellani, Georg Singer, Holger Till

**Affiliations:** Department of Pediatric and Adolescent Surgery, Medical University of Graz, Auenbruggerplatz 34, 8036 Graz, Austria

**Keywords:** Medical research, Paediatric research

## Abstract

The aim of this study was to assess whether adolescents following anorectal malformation repair have a decreased cardiorespiratory performance capacity and impaired motor skills. All eligible children treated for ARMs between 2000 and 2014 were invited to participate in a prospective study consisting of a clinical examination, evaluation of Bowel function and Quality of Life, spirometry, spiroergometry and assessment of the motor activity. The results were compared to a healthy age- and sex-matched control group. There was no statistically significant difference in height, weight, BMI, muscle mass or body fat percentage between the study and the control group. Nine out of 18 patients (50%) had an excellent functional outcome with a normal Bowel Function Score. Spirometry revealed no significant differences between ARM patients and controls, four patients showed a ventilation disorder. Spiroergometry revealed a significantly lower relative performance capacity and the overall rating of the motor activity test showed significantly decreased grades in ARM patients. ARM patients were affected by an impaired cardiopulmonary function and decreased motor abilities. Long-term examinations consisting of routine locomotor function evaluation and spiroergometry are advisable to detect impaired cardiopulmonary function and to prevent a progression of associated complications and related impaired quality of life.

## Introduction

Anorectal malformations (ARMs) are common congenital malformations occurring in 1 out of 2000 to 5000 live births and present with a broad spectrum of anatomical variants^1,2^. In up to 60% of the cases, ARMs are associated with spinal, genitourinary or skeletal anomalies. These lead to highly variable rates of urinary and fecal incontinence or constipation^2^. The clinical outcome depends—amongst others—on the type of the malformation, concurring spinal abnormalities and the sacral ratio^3^. Especially the sacral development significantly affects the functional prognosis for bowel function^3^.

Fecal continence and psychosocial problems are considered the most common active issue in teenagers born with ARMs^1^. Quality of Life (QoL) including social as well as physical functioning is significantly lower in children following ARM repair compared to controls^4^. Moreover, QoL of children with ARMs and poor fecal continence is even lower compared to children with ARMs and good fecal continence^4^. Soiling and constipation correlate significantly with psychosocial morbidity^5^. However, physical activity can enhance QoL in children with chronic health conditions^6^.

Long-term results regarding bowel function, fecal continence, QoL and sexual function following the repair of an ARM are well described in the literature^1,2,7^. However, examinations of the physical performance following ARM repair are lacking. Decreased cardiorespiratory performance and impaired motor skills frequently occur in patients with congenital malformations, such as esophageal atresia (EA) or congenital diaphragmatic hernia (CDH) and in children with chronic health conditions^6,8–10^.

Knowing that impaired sacral development and spinal defects can have a major impact on the functionality of the pelvis and lower extremities we evaluated if patients following ARM repair have a decreased cardiorespiratory performance capacity and impaired motor skills compared to a healthy age and sex matched control group. Additionally, we assessed the QoL and functional outcome in relation to the individual sports performance in ARM patients.

## Patients and methods

All consecutive eligible children treated for ARMs between 2000 and 2014 were invited to participate in a prospective study including clinical examination, spirometry, cardiopulmonary exercise performance testing (CPET) and assessment of the motor activity.

This study was performed according to the declaration of Helsinki. Informed written consent was obtained from all patients and controls and/or their legal guardians. The study was approved by the Ethics Committee of the Medical University of Graz (EK 31–338 ex 18/19) and followed the STROBE criteria.

The medical records of the ARM patients were retrospectively reviewed for associated congenital anomalies, disease-related co-morbidities and consecutive interventions. All patients were classified according to the Krickenbeck classification^11^. The results were compared to a healthy age- and sex matched control group recruited from the personal environment of the Department´s employees or patients.

### Anthropometric data

At the study visit, cardiac arrhythmias were excluded with a 12-lead resting electrocardiography (ECG) and non-invasive blood pressure (NIBP) measurement at rest was performed. Both height and body weight (BW) were assessed and the body mass index (BMI) Z-score calculated. The body fat in percent was determined applying the caliper method using a 4-site skin fold procedure and estimated according to a standardized table^12,13^.

Multi-frequency impedance spectroscopy (Combyn ECG, Academic Technologies at the Institute of Cardiovascular Medicine GmbH, Graz, Austria) was used to measure appendicular muscle mass as previously described in the literature^14^.

### Evaluation of fecal continence and quality of life (QoL)

All participants were asked to assess their fecal continence using the standardized bowel function score (BFS) developed by Rintala^15^. This questionnaire rates functional outcome following ARM repair and is validated on a population of healthy children^16,17^. It consists of seven questions, including the ability to hold back, feel urge to defecate, frequency of defecation, soiling, accidents, constipation and social problems^15^. Each question can be scored from 0 to 3, except the frequency of defecation, which is scored 1–2, leading to a BFS score ranging from 1 to 20. The clinical outcome was classified as excellent (score ≥ 18), good (12–17), fair (7–11) or poor (score ≤ 6) as described before^16^. Only patients with a score of at least 18 were classified as having normal bowel function^16^.

The QoL was assessed according to the “Quality-of-Life Scoring Criteria for Children with Fecal Incontinence” published by Bai and coworkers asking for soiling, incontinence, school absence, unhappiness or anxiety, food restrictions and peer rejections^4^. This scoring system ranges from 2 to 13 with higher scores describing a better QoL^4^.

### Spirometry

Small spirometry (Oxycon Pro, Carl Reiner GmbH, Vienna, Austria) at rest and following exercise was used to measure lung function. Maximum vital capacity (VC_max_) and the forced expiratory volume in 1 s (FEV 1) were determined. VC_max_ was expressed as observed over age and gender and corrected according to the expected maximum vital capacity. The Tiffeneau index was calculated as FEV 1/VC_max_. An obstructive ventilation disorder was diagnosed by a decreased Tiffeneau index, a restrictive pattern was suggested by predominantly decreased VC_max_^18^. Following the cardiopulmonary exercise performance testing spirometry examination was repeated in order to rule out exercise-induced asthma by showing a decreased Tiffeneau index after intense physical activity^19^.

### Cardiopulmonary exercise performance testing (CPET)

CPET with a bicycle ergometer (Excalibur Sport, Lode B.V., Groningen, The Netherlands) and the spirometer in an upright position was used to measure cardiopulmonary exercise performance. A gender and age adapted protocol using a stepwise load increase as previously published was applied^13^. The spiroergometry was carried out to subjective exhaustion or until the participants were unable to maintain the required pedaling speed (cadence) of more than 60 revolutions per minute (rpm). A 3 min recovery period of slow pedaling (60 rpm) with the same workload as at the beginning of the test followed the exercise phase. Heartrate (HR) was measured by continuous twelve-lead ECG (Combyn ECG, Academic Technologies at the Institute of Cardiovascular Medicine GmbH Graz, Austria). Oxygen saturation was continuously assessed (Finger Pulse Oximeter Habel Medizintechnik, Vienna, Austria). Lactate levels were obtained by earlobe sampling of 20 µl blood per measurement to heparinized capillaries before the test, at the end of each step and after the recovery phase (enzymatically amperometric measurement with a Biosen C_line (EKF Diagnostics for Life, Cardiff, UK)).

The respiratory parameters included the oxygen uptake (VO_2_), the oxygen pulse (O_2_/HR), the respiratory equivalent for oxygen (EQO_2_), the breathing reserve (BR), the aerobic capacity (ΔVO_2_/ΔWR) and the respiratory exchange ratio (RER)^13^.

Relative performance capacity was calculated from the achieved maximal wattage in relation to age and gender-specific standard values^20^. The peak oxygen uptake (peak VO_2_) was defined as the average VO_2_ over the last 30 s prior to subjective exhaustion and was expressed in ml/kg/min. A RER > 1.10 was used as criterion to determine that the peak VO_2_ reflects a peak physiological workload^21^.

### Assessment of motor abilities

Motor abilities were assessed using the Dordel–Koch-Test (DKT) as a test for the assessment of flexibility, coordinative and conditional skills in children and adolescents^22^. The tests consists of seven established and validated items: lateral jumping, sit and reach, sit-ups, long stand jump, one-legged stand, push-ups and 6-min-run and allows a quick and differentiated evaluation of motor performance among all basic motor skills^22^. In the present study, the endurance was tested with a spiroergometry instead of a 6-min-run. The indicated grades 1 to 6 correspond to the school grading system with lower values indicating better performance^22^.

### Statistical analysis

All data were entered into an Excel 2019 spreadsheet. SPSS Statistics 21 (IBM Corp. Released 2012. IBM SPSS Statistics for Windows, Version 21.0. Armonk, NY: IBM Corp) was used for statistical analysis. While continuous data are displayed as mean and standard deviation, categorical data are presented as numbers and/or percentages.

A Kolmogorov Smirnov Test was used to assess normal distribution and Levene´s Test to assess the homogeneity of variances. In case of normal distribution and homogeneity of variances, statistical group comparison was performed using a 2-sided, unpaired t‐test. In case of absent normal distribution and homogeneity of variances, a Mann–Whitney-U-Test was used for group comparison. Categorical data were compared with the Chi-squared Test.

Correlation analysis was performed with Pearson correlation tests. Additionally, multiple linear regression analyses were run to identify factors that are associated with relative performance capacity and results of the DKT.

Explorative statistical significance was defined as *p* < 0.05.

## Results

Out of 27 eligible patients with an ARM, 21 agreed to participate. At the follow-up examinations, three patients had to be excluded because of incompliant spirometry and spiroergometry, leaving 18 patients for further analysis. One of the included patients was initially operated in an external hospital.

### ARM group

The mean age of the ARM group (13 females, 5 males) was 13.6 ± 3.0 years (range: 9–18 years). The different types of ARM and surgical repair are listened in Table 1.

**Table 1:** ARM patients, age at time of follow-up, type of malformation, surgery, BFS according to Rintala^23^, QoL according to Bai et al.^4^ and functional outcome.

ID	Age	Gender	Type	Additional anomaly	Surgery	Rintala	Bai
Pat.1	9	f	Perineal fistula	–	PSARP^a^	19	10
Pat.2	9	f	Perineal fistula	–	PSARP^a^	17	12
Pat.3	9	f	Perineal fistula	–	PSARP^a^	15	11
Pat.4	11	f	Perineal fistula	–	PSARP^a^	20	12
Pat.5	11	f	Rectovestibular fistula	Anophthalmus and duodenal atresia	PSARP^a^	20	11
Pat.6	13	f	Perineal fistula	–	PSARP^a^	17	13
Pat.7	14	f	Rectovestibular fistula	–	PSARP^a^	16	13
Pat.8	15	f	Perineal fistula	–	PSARP^a^	18	13
Pat.9	15	f	Perineal fistula	–	PSARP^a^	20	13
Pat.10	15	f	Rectovestibular fistula	Currarino triad	PSARP^b^	13	12
Pat.11	16	f	Rectovestibular fistula	–	PSARP^a^	20	13
Pat.12	16	f	Perineal fistula	–	PSARP^a^	9	10
Pat.13	17	f	Cloaca	EA, pancreas anulare, volvulus	Abd.-perineal pull-through	Colostomy	Colostomy
Pat.14	10	m	Imperforate anus	–	PSARP^a^	20	12
Pat.15	13	m	Perineal fistula	–	PSARP^b^	18	13
Pat.16	16	m	Perineal fistula	–	PSARP^a^	19	13
Pat.17	17	m	Rectovesical fistula	Caudal regression	PSARP^b^	15	9
Pat.18	18	m	Rectobulbar fistula	–	PSARP^b^	11	9

^a^One-staged procedure (PSARP).

^b^Three-staged procedure (colostomy, PSARP, colostomy closure).

Thirteen patients (72.2%) received a single-stage repair (posterior sagittal anorectoplasty, PSARP). The remaining five patients (27.8%) were treated with a three-staged surgical procedure consisting of neonatal diverting colostomy, PSARP and colostomy closure (Table 1).

Spinal anomalies were documented in two patients: One girl presented with a Currarino triad, consisting of an ARM with a rectovestibular fistula, a ventral myelomeningocele (MMC) and a partial sacral agenesis. The other patient had a caudal regression syndrome with agenesis of the os coccygis and fused 4th and 5th sacral vertebrae and was operated in an external hospital.

Another two female patients had at least one additional congenital anomaly: One girl suffered from an EA and a pancreas anulare and had to be operated because of a volvulus. The remaining patient was born with a unilateral anophthalmus and a duodenal atresia.

The following post-operative complications were recorded: One patient (5.6%) had an anal stenosis and the externally operated patient presented with an iatrogenic urethral lesion and a vesico-ileo-cutaneostomy.

### Control group

The mean age of our age and sex matched control group was 13.3 ± 3.1 years (range: 8–18 years) and consisted of 13 female and 5 male participants. There was no statistically significant difference regarding patient age between ARM and control groups (*unpaired t-test*; *p* = *0.828).*

### Anthropometric data

There was no statistically significant difference in height, weight, BMI Z-score, muscle mass or body fat percentage between the study and the control group (Table 2).

**Table 2:** Anthropometric data, results of spirometry and spiroergometry and Dordel-Koch-Test (DKT) of ARM patients and controls (n = 18 each).

	ARM patients	Controls	*p*-value
	n = 18	n = 18	
Age	13.6 ± 3.0	13.3 ± 3.1	0.828 $
Gender (male/female)	13/5	13/5	1.000
**Anthropometry**			
Height [m]	1.6 ± 0.1	1.6 ± 0.2	0.419 $
Body weight [kg]	49.7 ± 13.4	47.3 ± 15.8	0.636 $
BMI Z-score	− 1.6 ± 5.9	− 0.34 ± 1.1	0.913
Body fat [%]	15.1 ± 8.1	11.1 ± 7.3	0.134 #
Muscle mass [kg/height^2^]	5.5 ± 1.7^a^	6.0 ± 2.1	0.425 $
**Spirometry**			
VC_max_ [%]	93.9 ± 13.3	102.0 ± 12.5	0.069 $
Tiffeneau Index [%]	86.6 ± 10.2	87.7 ± 5.2	0.695 $
**Spiroergometry**			
Relative performance [%]	110.4 ± 19.2	127.8 ± 16.9	**0.007 $**
Peak VO_2_ [ml/kg/min]	41 ± 8.2	45.6 ± 9.4	0.323 #
O_2_/HR [ml]	10.7 ± 3.4	10.8 ± 4.1	0.927 $
EQO_2_	20.7 ± 3.1	20.2 ± 2.5	0.557 $
BR	15.0 ± 14.9	19.7 ± 16.0	0.367 $
ΔVO_2_/ΔWR	11.4 ± 1.2	11.1 ± 1.3	0.444 $
**Dordel–Koch test (DKT)**			
Lateral jumping	4.1 ± 1.1	2.6 ± 1.1	** < 0.001 #**
Sit and reach	3.6 ± 1	3.2 ± 0.9	0.279 #
Sit-ups	3.7 ± 0.9	2.7 ± 0.8	**0.008 #**
Long stand jump	4.1 ± 1.1	2.8 ± 0.9	**0.001 #**
One-legged stand	2.2 ± 1.8	1 ± 0	0.091 #
Push-Ups	2.7 ± 1.1	1.9 ± 0.8	0.059 #
**DKT**	**3.4 ± 0.7**	**2.4 ± 0.5**	** < 0.001 #**

Bold values indicate statistically significant differences.

All data are displayed as mean ± standard deviation and statistical comparison was performed using either unpaired t-tests ($) or Mann–Whitney-U tests (#) depending on normal distribution and homogeneity of variances.

^a^Measurement of muscle mass was technically not possible in one control patient.

### Evaluation of fecal continence and quality of life (QoL)

Nine out of 18 patients (50%) had an excellent functional outcome with a normal BFS, comparable to healthy children. A good result was achieved in 6 patients (33.3%). Two patients (11.1%) had a fair clinical outcome (BFS 9 and 11). One female patient with a cloaca still has her colostomy following abdomino-perineal pull through procedure and denies further surgeries, she was not included in the BFS score (Table 1).

The BFS did not correlate significantly with the cardiorespiratory performance capacity (*r* =  − *0.055, p* = *0.834*) or results of the DKT (*r* = *0.075, p* = *0.775*).

The Quality of Life was good in the majority of our patients with a mean QoL of 11.7 ± 1.4 (range 9–13). The QoL correlated significantly with the BFS (*r* = *0.559, p* = *0.02*).

### Spirometry

Spirometry revealed no significant differences of VC_max_ or Tiffeneau index (Table 2). Four patients showed a ventilation disorder, which occurred only in the ARM patient group. One had a restrictive, two a combined and another one an obstructive ventilation disorder. Two patients with a ventilation disorder had an additional congenital anomaly (esophageal atresia and a duodenal atresia).

### Cardiopulmonary exercise performance

The RER value (ARM mean 1.1 ± 0.1 vs. controls mean 1.1 ± 0.1) did not differ significantly between the two groups (*unpaired t-test, p* = *0.839)*. Spiroergometry revealed a significantly lower relative performance capacity (*unpaired t-test; p* = *0.007*) in the ARM group compared to the control group (Supplementary Fig. S1). Peak VO_2_, O_2_/HR, EQO_2_, BR and ΔVO_2_/ΔWR did not show any significant differences (Table 2). Following removal of the five patients who underwent three-staged procedures and their respective controls, the relative performance capacity remained significantly different between the two groups (ARM mean 109.2 ± 20.5 vs. controls mean 128.4 ± 17.6; *p* = 0.027, unpaired T-test). The remaining spirometry and cardiopulmonary exercise performance test values following removal of these children are shown in Supplementary Table S1.

A multiple regression was run to predict relative performance capacity from gender, BMI Z-score, body fat, muscle mass, VC_max_, Tiffeneau Index, peak VO_2_, EQO_2_, BR, ΔVO_2_/ΔWR and DKT. These variables statistically significantly predicted relative performance capacity, *F*(11, 23) = 13.998, *p* < 0.001, *R*^*2*^ = 0.87. Gender, Tiffeneau Index, peak VO_2_ and ΔVO_2_/ΔWR added statistically significantly to the prediction, *p* < 0.05.

### Assessment of motor abilities

The overall rating of the DKT showed significantly worse grades in the ARM group (*Mann–Whitney-U-test; p* < *0.001*); significant differences were found in lateral jumping, sit-ups and long stand jump (Table 2). Even after removal of the five patients with a three-staged procedure and their respective controls, the overall rating of the DKT remained significantly different in the ARM patients compared to controls (ARM mean 3.4 ± 0.7 vs. controls mean 2.3 ± 0.5; *p* < 0.001, Mann–Whitney-U test).

A multiple regression was run to predict results of the DKT from gender, BMI Z-score, body fat, muscle mass, VC_max_, Tiffeneau Index, EQO_2_, BR, ΔVO_2_/ΔWR and relative performance capacity. These variables statistically significantly predicted results of the DKT, *F*(10, 24) = 3.091, *p* < 0.011, *R*^2^ = 0.563. Only the relative performance capacity added statistically significantly to the prediction, *p* < 0.05.

The raw data of our findings are listed in Supplementary Table S2.

## Discussion

This study presents parameters of lung function, exercise and motor performance in combination with a mean follow-up of 13 years in ARM patients. The main findings of the present study were a significantly decreased exercise performance as well as significant deficits in motor abilities in ARM patients compared to healthy age and sex matched controls.

The majority of our patients had specific anatomical variants of ARM with a good prognosis (recto-perineal fistula, recto-vestibular fistula, imperforate anus with normal sacrum). In 15 out of our 18 patients an excellent or good bowel function could be achieved. However, normal bowel habits, defined as BFS ≥ 18^23^, were seen in only 50% of the patients (see Table 1). Comparable results were described in previously published studies, where the functional outcome of ARM patients with a good prognosis were not comparable to healthy children^16,17^.

In the present study, five patients with a neonatal colostomy were included; four of them showed a decreased BFS at follow-up. It is known from the literature that a neonatal colostomy is associated with a worse functional outcome compared to children who undergo a primary repair^16^. The underlying reason, however, remains unclear, but defunctionalisation of the recto-sigma combined with a non-activation of the so called “brain-defecation reflex” have been hypothesized^16^. However, exclusion of the patients with a three-staged procedure and their respective controls did not lead to different results, showing that additional abdominal surgeries with forming and closure of colostomy did not significantly influence the cardiorespiratory performance capacity or motor abilities.

Spirometry revealed no significant long-term pulmonary impairment of ARM patients. Nevertheless, ventilation disorders occurred only in the ARM patient group (n = 4). Two of these were born with an additional congenital anomaly. In one patient, the combined ventilation disorder was most likely related to a concurring EA, in which long-term pulmonary impairment and reduced lung volumes are frequently found^24,25^.

Patients following ARM repair showed a significantly lower relative performance capacity compared to controls in bicycle spiroergometry. This could be associated with a decreased physical fitness and impaired locomotor function as previously described in patients with other congenital anomalies such as EA and CDH or chronic health conditions^6,8–10,26^. Recently reported long-term problems following ARM repair show prevalence of fecal incontinence ranging from 16.7 to 76.7% and chronic constipation occurring in 22.2% to 86.7% of affected patients^2^. Given these numbers, a significant proportion of ARM patients seem to have major restrictions in their social lives. Thus, participation in sports and social activities may be frequently restricted and may result in social disability and reduced physical performance. However, in our patient cohort there was no significant correlation between the functional outcome and the cardiopulmonary performance capacity or motor abilities. Nevertheless, a routine locomotor function evaluation in school-aged ARM patients seems to be advisable even in patients with an excellent outcome.

Items of the DKT related to the lower extremities such as lateral jumping and long stand jump were significantly worse in ARM patients (compare Table 2). This fact may be a part of disease-related sequelae and probably be related to an impaired functionality of the pelvis, sacrum and lower extremities. However, only two of our patients had a sacral anomaly indicating that even in normal spinal development some kind of impairment of the lower extremities may persist. Furthermore, Arnoldi and coworkers have proposed that also in patients with a normal sacrum and a normal sacral ratio a complete screening for neurospinal anomalies is mandatory, even in ARMs with a good prognosis^16^.

The QoL significantly correlated with the functional outcome as previously published^4,5^. QoL consists of social and physical functioning of the child and can be severely impaired by fecal incontinence. Soiling and odor are socially unacceptable, leading to internalizing, depression and restriction of social activities^4^. In a long-term follow up with a mean age of 10 years, 80% of ARM patients had behavioral problems and 15% suicidal thoughts. Moreover, the psycho-social morbidity correlated significantly with soiling occurring in 60% of patients^5^. Thus, preventing social disability is a fundamental part of the postoperative management in ARM patients and demands a clear and structured bowel management program. Furthermore, a defined transitional care and multidisciplinary review must be offered in every institution to continue the medical care in adulthood^7^.

Our results concerning motor abilities warrant further ideally multi-centric studies including a larger group of patients with additional methods such as the Bruininks-Oseretsky Test of Motor Proficiency^27^ delivering a more precise and comprehensive measure of both gross and fine motor skills in order to confirm our findings and facilitate a more specific differentiation of the kind of motor impairment. Moreover, it would be interesting to examine whether targeted physiotherapy in the follow-up care can lead to an improvement in the basic sports motor properties of ARM patients.

### Limitations

A possible limitation of this study is the relatively small sample size. However, large sample sizes in orphan pediatric diseases are difficult to achieve. The strengths of our study are the long-term follow up of 13 years and the inclusion of an age- and sex-matched control group.

## Conclusion

Even ARM patients without spinal abnormalities were affected by an impaired cardiopulmonary function and decreased motor abilities. Long-term examinations of patients with ARM consisting of routine locomotor function evaluation and spiroergometry are advisable and necessary in order to detect impaired cardiopulmonary function and to prevent a progression of associated complications and related impaired QoL. The long-term pulmonary function following ARM repair, especially in patients with additional congenital anomalies, should be investigated in prospective, multi-center studies.

## Supplementary Information

Supplementary Legends.

Supplementary Figure S1.

Supplementary Table S1.

Supplementary Table S2.
