# Supplementary material for: Cardiorespiratory performance and locomotor function of patients with anorectal malformations
Source: Sci Rep. 2021 Sep 23;11:18919. doi: 10.1038/s41598-021-98368-z (PMC8460638; doi:10.1038/s41598-021-98368-z)
Supplement: Supplementary file 1 — Supplementary Legends. [file 41598_2021_98368_MOESM1_ESM.docx]

**Supplementary Figure and Table Legends**

**Supplementary Figure S1:** Performance capacity of ARM patients compared to controls (*…*p=0.007, unpaired t-test*).

**Supplementary Table S1:** Anthropometric data, results of spirometry and spiroergometry and Dordel-Koch-Test (DKT) of ARM patients without three-staged operations and their respective age- and sex-matched controls (n=13 each). All data are displayed as mean ± standard deviation and statistical comparison was performed using either unpaired t-tests ($) or Mann-Whitney-U tests (#) depending on normal distribution and homogeneity of variances; ^a^…measurement of muscle mass was technically not possible in one control patient.

**Supplementary Table S2:** Raw Data.
