# Supplementary figures and images for: Cardiorespiratory performance and locomotor function of patients with anorectal malformations
Source: Sci Rep. 2021 Sep 23;11:18919. doi: 10.1038/s41598-021-98368-z (PMC8460638; doi:10.1038/s41598-021-98368-z)

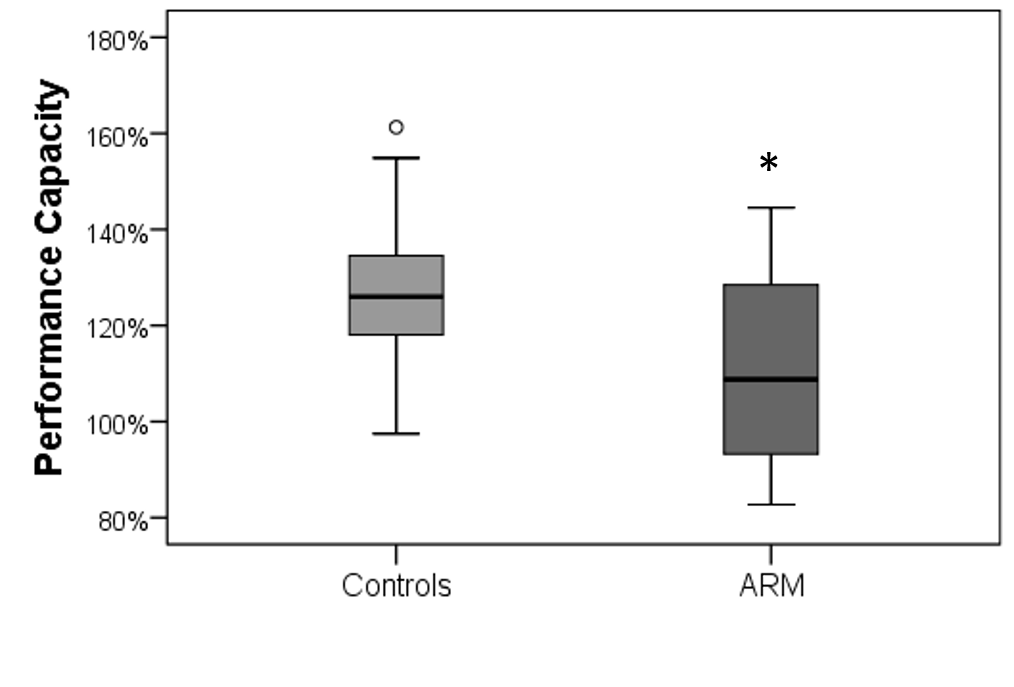

Supplement: Supplementary file 2 — Supplementary Figure S1. [file 41598_2021_98368_MOESM2_ESM.tif]
