# Supplementary material for: Cardiorespiratory performance and locomotor function of patients with anorectal malformations
Source: Sci Rep. 2021 Sep 23;11:18919. doi: 10.1038/s41598-021-98368-z (PMC8460638; doi:10.1038/s41598-021-98368-z)
Supplement: Supplementary file 3 — Supplementary Table S1. [file 41598_2021_98368_MOESM3_ESM.docx]

|  | **ARM patients** | **Controls** | **p-value** |
| --- | --- | --- | --- |
|  | **n=18** | **n=18** |  |
| **Age** | 12.6 ± 2.9 | 12.8 ± 2.8 | 0.84 # |
| **Gender (male/female)** | 11/2 | 11/2 | 1.000 |
| **Anthropometry** |  |  |  |
| Height [m] | 1.6 ± 0.1 | 1.5 ± 0.2 | 0.564 $ |
| Body Weight [kg] | 48.7 ± 15.6 | 46.7 ± 17.3 | 0.576 $ |
| BMI Z-score | 0.09 ± 0.7 | -0.16 ± 1.2 | 0.418 # |
| Body Fat [%] | 17 ± 7.5 | 12.5 ± 7.4 | 0.091 # |
| Muscle Mass [kg/height²] | 5.1 ± 1.6^a^ | 5.5 ± 2.1 | 0.673 $ |
| **Spirometry** |  |  |  |
| VC_max_ [%] | 95.6 ± 12 | 98.8 ± 9.9 | 0.643 $ |
| Tiffeneau Index [%] | 88.7 ± 7.3 | 88.6 ± 4.9 | 0.956 $ |
| **Spiroergometry** |  |  |  |
| Relative Performance [%] | 109.2 ± 20.5 | 128.4 ± 17.6 | **0.027 $** |
| peak VO_2_ [ml/kg/min] | 39.3 ± 6.6 | 44.8 ± 9.4 | 0.511 # |
| O_2_/HR [ml] | 9.9 ± 3.2 | 10.2 ± 3 | 0.934 $ |
| EQO_2_ | 20 ± 3.1 | 20.1 ± 2.3 | 0.448 # |
| BR | 17.9 ± 16.4 | 20.1 ± 17.2 | 0.618 $ |
| ∆VO_2_/∆WR | 11.6 ± 1.3 | 11.2 ± 1.5 | 0.362 $ |
| **Dordel-Koch Test (DKT)** |  |  |  |
| Lateral Jumping | 3.9 ± 1.2 | 2.3 ± 1.1 | **0.001 $** |
| Sit and Reach | 3.7 ± 1 | 3 ± 0.9 | 0.125 # |
| Sit-Ups | 3.6 ± 1 | 2.6 ± 0.9 | **0.016 #** |
| Long Stand Jump | 4 ± 1.1 | 2.8 ± 0.9 | **0.019 #** |
| One-legged Stand | 2.4 ± 1.9 | 1 ± 0 | 0.101 # |
| Push-Ups | 2.6 ± 1.2 | 1.9 ± 0.9 | 0.101 # |
| **DKT** | **3.4 ± 0.7** | **2.3 ± 0.5** | **<0.001 #** |
|  |  |  |  |

Supplementary Table 2: Anthropometric data, results of spirometry and spiroergometry and Dordel-Koch-Test (DKT) of ARM patients without three-staged operations and their respective age- and sex-matched controls (n=13 each). All data are displayed as mean ± standard deviation and statistical comparison was performed using either unpaired t-tests ($) or Mann-Whitney-U tests (#) depending on normal distribution and homogeneity of variances; ^a^…measurement of muscle mass was technically not possible in one control patient.
